# Bilateral massive osteolysis of uncertain origin after total knee arthroplasty: A case report and review of literature

**DOI:** 10.1016/j.ijscr.2021.105678

**Published:** 2021-02-26

**Authors:** Rachid Rassir, Jorm M. Nellensteijn, Rachid Saouti, Peter A. Nolte

**Affiliations:** a Spaarne Gasthuis, Spaarnepoort 1, 2134 TM Hoofddorp, the Netherlands; b Amsterdam UMC, Location VUmc, De Boelelaan 1117, 1081 HV Amsterdam, the Netherlands

**Keywords:** Case report, Total knee arthroplasty, Periprosthetic osteolysis, Revision total knee arthroplasty, Metaphyseal bone loss, Allograft bone impaction

## Abstract

**Introduction and importance:**

Periprosthetic osteolysis (PPOL) is a common complication after total knee arthroplasty (TKA) and is most commonly caused by wear-induced particles.

**Case presentation:**

We report an unusual case of massive bilateral PPOL in the posterior flanges of the femur and patellae 4 years after bilateral uncemented TKA without patellar resurfacing in a 71-year old female. Bilateral staged revision surgery including polyethylene exchange and allograft morselized bone impaction was performed to treat the osteolytic lesions. There were no signs of implant malalignment, polyethylene wear or component loosening.

**Clinical discussion:**

Several factors are associated with an increased risk on PPOL (e.g. polyethylene sterilization method, patient age, male gender). Surgical intervention in the context of massive PPOL should include replacement of a potential particle generator (most often polyethylene), correction of potential malalignment, treatment of bone defects and assessment of implant anchorage.

**Conclusion:**

This report highlights the available evidence on clinical presentation, associated risk factors and preferred treatment strategy of massive osteolytic lesions after TKA according to available evidence.

## Highlights

• Osteolysis can occur in the patella without a patellar prosthesis.
• Increased shelf-age and lower sterilization dose of inserts predispose for osteolysis.
• Conventional radiographs lack sensitivity in detecting and monitoring osteolysis.
• Insert exchange, implant anchorage/alignment and bone defects are surgical priorities.
• Although bone defects can be massive, no total revision is needed with good anchorage.

## 1 Introduction

Periprosthetic osteolysis (PPOL) is a common complication after total joint arthroplasty [1]. It is most often caused by wear-induced particles from the implant components (polyethylene, metal debris) triggering a chronic-inflammatory reaction that stimulates osteoclastic activity and suppresses bone formation [2,3]. There is also strong evidence for the role of mechanical forces in the development of PPOL [[4], [5], [6]], possibly being the primary causative factor that leads to inflammatory activity at the prosthesis-bone interface [7]. Various other reasons for the occurrence of PPOL are proposed in the literature, such as low grade infection, malrotation or loosening of the prosthesis or an allergy to the inserted material [2].

Massive osteolysis mostly occurs in the posterior flange of the femur and along access tracks of the tibial component [8]. PPOL is rarely seen in the patella without a patellar component in situ [9,10]. Modern TKA systems have addressed the problem of wear-related osteolysis by the development of highly crosslinked thermally treated polyethylene bearings and vitamin-E infusion [11,1]. We present a case of idiopathic massive bilateral PPOL of both patellae and femora after uncemented TKA, without any apparent signs of advanced wear of the components. We furthermore review the available evidence regarding diagnosis, risk factors and surgical treatment modalities of massive osteolytic lesions after TKA.

## 2 Case presentation

The current case report has been reported in line with the 2020 Consensus Surgical CAse REport (SCARE) guidelines [12]. A 71-year old female was referred to the orthopedic outpatient clinic in our hospital with progressive pain in both knees. Four years earlier, a mobile bearing uncemented TKA without patellar resurfacing (Low Contact Stress; DePuy Synthes, Warsaw, IN, USA) had been implanted in both knees due to debilitating osteoarthritis. No complications occurred peri-operatively, but pain and stiffness of both knees was present since surgery. Besides hypertension, the patient had no further medical history.

**Fig. 1:**
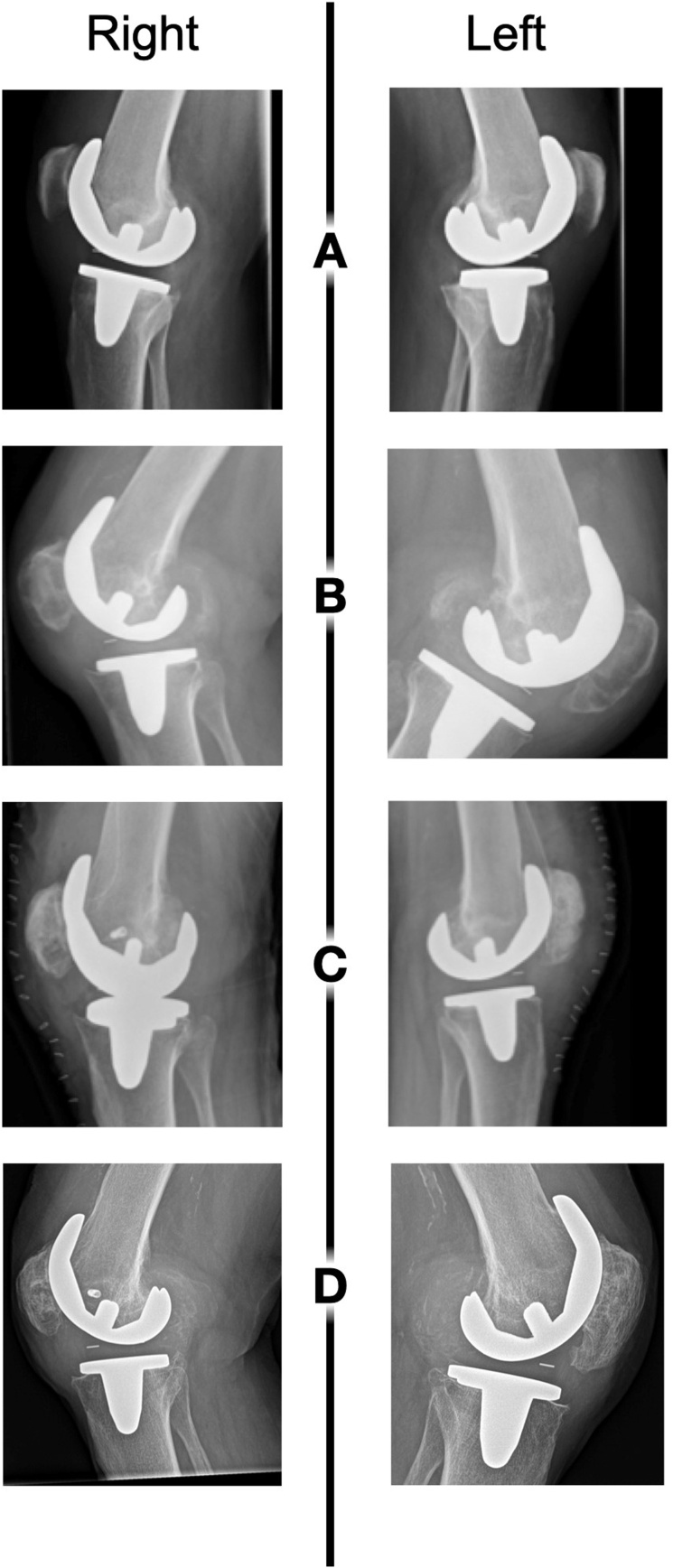
Radiographic diagnosis and follow-up of the massive osteolytic lesions. (A) radiographs after initial index TKA surgery, demonstrating acceptable alignment and no preexistent bone deficiencies; (B) 4 years post index TKA surgery the radiographs showed massive osteolytic lesions in the posterior aspect of the femur and center of the patella in both knees; (C) directly after revision, with allograft bone impaction grafting and collateral ligament fixation (right) visible; (D) 8 years post revision and treatment of the osteolysis, showing no relapse or development of new lesions.

**Fig. 2:**
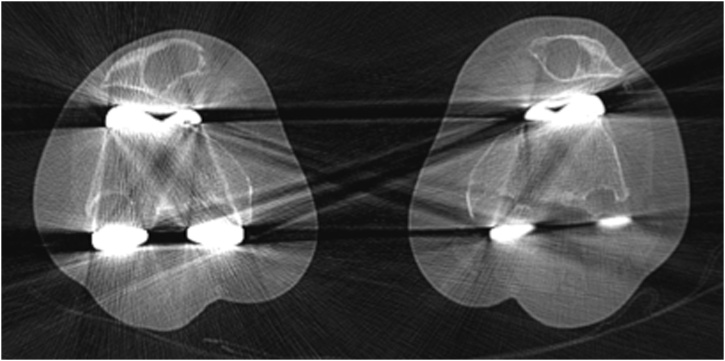
Computed tomography slices of both knees (left is right and right is left) at the level of the femoral metaphyses. Severe bone loss is visible at the posterior aspect of the femur and patella.

Physical examination revealed warm, swollen knees, flexion/extension of 90/0/0° and pain on palpation of the joint lines. The implanted knees were stable in coronal and sagittal planes. Conventional radiographs and computed tomography scans showed a similar pattern of extensive osteolytic lesions in the posterior femoral condyle and center of the patella in both knees (Figs. 1 & 2). Blood tests were normal, with a C-reactive protein of < 2,5 mg/L (normal < 8 mg/L) and WBC of 9,3 × 10^9^/L (range 4–10 × 10^9^/L). Allergy tests for metals by our hospital allergist were negative. Knee aspiration cultures excluded infection.

**Fig. 3:**
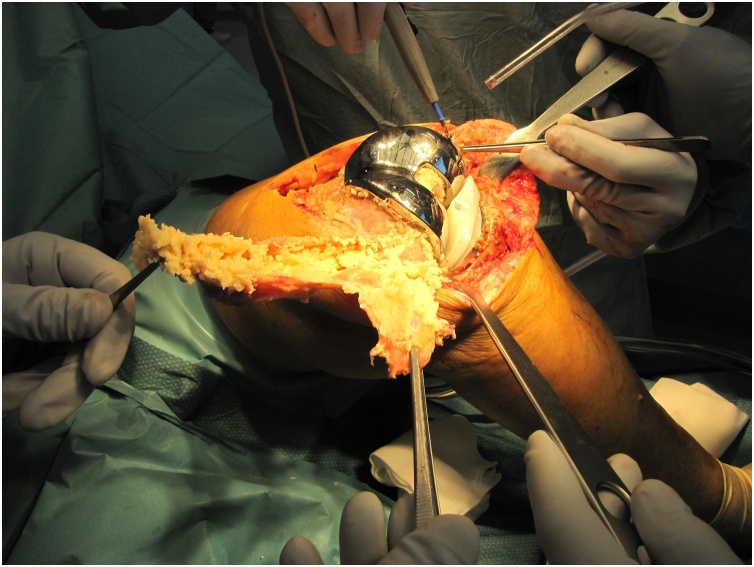
After arthrotomy and exposure of the knee joint, an extensive caseous synovitis was revealed during revision surgery of the left knee.

**Fig. 4:**
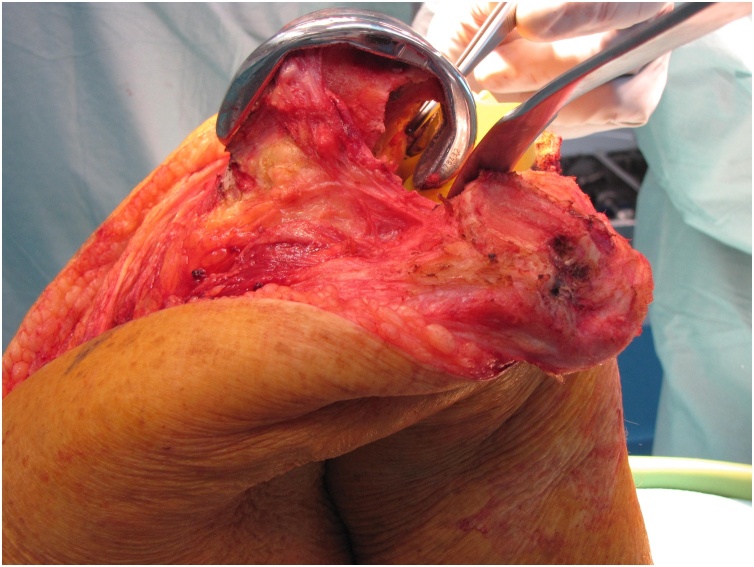
Lateral photograph depicting the severity of the bone loss in the femur.

**Fig. 5:**
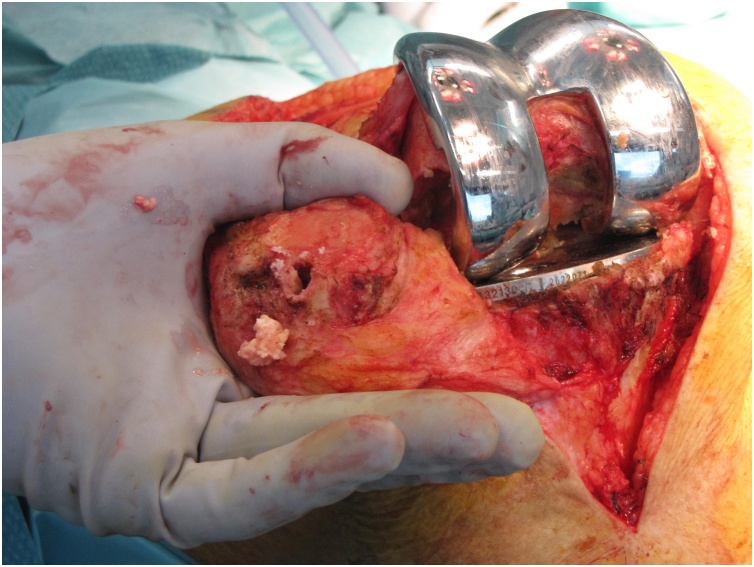
Osteolysis found in the center of the articulating surface of the patella during revision surgery of the right knee.

Revision surgery of both knees, starting with the right knee with an interval of four months, was performed by 2 orthopedic surgeons (RS, PN, both extensive experience with revision TKA). After medial parapatellar arthrotomy a caseous synovitis was visible (Fig. 3). An extensive synovectomy was performed. There was no malalignment and there was firm anchorage to the bone of both uncemented femoral and tibial components. The posterior part of the lateral condyle was absent in both knees, classified as a type 2A bone defect according to the Anderson Orthopedic Research Institute (AORI) system (Fig. 4) [13]. Only slight delamination could be detected on the polyethylene bearings. Because there was good fixation of the femoral component on the anterior part of the femur, only the insert was exchanged and the posterior defect was filled by allograft bone impaction, using a morselized femoral head from the hospital bone bank. In the center of the articular side of the patella a contained defect was visible, without severe osteolysis of the posterior part of the patella (Fig. 5). This patellar defect contained necrotic tissue in both knees. The patellar defects were also treated with allograft bone impaction. In the right knee, the lateral collateral ligament was insufficient due to the missing part of the condyle and was re-attached with an anchor to the remaining part of the condyle. No complications were reported and the patient was able to mobilize (under direct supervision of the physical therapist) directly post-surgery. After revision surgery of the right knee, the patient was restricted to 90° knee flexion with a brace for 6 weeks because of the re-attachment of the lateral collateral ligament. After revision surgery of the left knee, the patient was allowed full weight bearing immediately. A low molecular weight heparin was prescribed as prevention of thrombosis for 6 weeks after both revision procedures. Post discharge, the patient was followed-up closely at the outpatient clinic.

Multiple tissue cultures from both knees revealed no microorganisms. Auramine colouring and DNA-PCR screening were both negative. Microscopic examination of the samples showed partly amorf eosinophilic material, an inflammatory reaction and birefringent material. At 8 years post revision surgery (12 years post primary TKA), the patient was pain free and able to mobilize normally with an unrestricted range of motion (110° of flexion with no extension lag in both knees). Radiographically, there was no new lesion of PPOL observed during the complete follow-up period (Fig. 1). After consultation by the rheumatologist and the internist to rule out any systemic causes for the PPOL, there was still no reason for the PPOL.

## 3 Discussion

PPOL is a common complication after TKA and is traditionally associated with polymethylmethacrylate (i.e. bone cement) debris [[14], [15], [16], [17]] and later became a problem of polyethylene wear and particles predominantly seen in uncemented implants [[18], [19], [20]]. Nowadays, polyethylene manufacturing has improved significantly and wear has markedly decreased as a reason of TKA failure [21]. Aseptic loosening, however, is the most common cause for revision TKA surgery [21], and in many cases PPOL precedes aseptic loosening [2]. PPOL is most commonly caused by wear-related polyethylene debris, but can also be caused by infection or metal allergy [2], the latter two have been ruled out as causes in our case. Even though macroscopic abrasion or delamination have not been observed during revision surgery in our case, subtle malalignment may still be present and generate a small amount of debris that caused a massive (hypersensitive) osteolytic reaction in the femur and patella. At the latest follow-up (12 years post index TKA), however, there are still no signs of relapse or development of new osteolytic lesions due to persisting malalignment, which makes prosthesis malalignment and generation of wear particles less likely to be the original culprit. Birefringent material in the synovia observed during histological examination may also be a sign of calcium pyrophosphate dihydrate crystal deposition disease (CPPD or pseudogout), which has been reported as a cause of arthritis even after TKA surgery [22]. However, CPPD is not known to cause massive osteolytic lesions and is therefore not the plausible cause of the massive osteolytic lesions observed in our case.

Osteolysis of the patella is quite common in TKA after placement of a patella component [23]. In our case, however, there was PPOL in the patella without placement of a patellar prosthesis, which is rarely reported in the literature. We only found 2 earlier reports of this observation after TKA [9,10]. Thomas et al. report on a case in which the patella appears to slowly disappear 5 years after TKA, without any symptoms or other lesions [10]. The authors speculate malalignment of the femoral component, and therefore accelerated polyethylene wear, to be a possible cause of the unusual sequel of events, but since no revision surgery was performed this cannot be proven [10]. Hassenpflug et al. describe an observational study of 206 knees receiving the Blauth knee system without patellar resurfacing [9]. In this cohort, 25 knees (12%) developed osteolytic lesions of the articulating part of the patella without any clear explanation given by the authors [9].

Several factors that increase the risk of PPOL have been identified through the years, such as screw fixation [24], specific patterns of porous coating (in uncemented implants) [25], modular components (versus monoblock) [26], ‘flat’ inserts (versus ‘deep-dished’) [27], lower sterilisation dose of inserts [[27], [28], [29]], shelf age of insert [27,29], cruciate retaining implants [27], biomechanical changes (posterior femoral condylar offset and joint line replacement) [30], patient age [27] and male gender [27]. Numerous implant manufacturers have attempted to address the implant-related concerns by improving upon existing knee designs. Besides the development of (thermally treated) highly crosslinked polyethylene [1,11] and improved polyethylene sterilisation methods [28], introduction of mobile bearings in TKA was one of these attempts to significantly reduce polyethylene wear and therefore PPOL [31]. These designs (e.g. the LCS the patient in our report received) feature a polyethylene liner that allows axial and/or anteroposterior rotation on the tibial component, theoretically providing lower contact stresses during knee kinematics and therefore less polyethylene wear [31]. Clinical translation of these proposed advantages over conventional, fixed bearing inserts are yet to be demonstrated. A comparison between wear-related revisions of mobile and fixed bearing implants even showed more osteolysis in the mobile bearing group [32], but this analysis included numerous designs and the discrepancy may be explained by design related differences between groups. Kim et al. randomized young patients undergoing bilateral TKA to one mobile and one fixed bearing TKA and found no differences between groups regarding osteolysis [33]. Likewise, other comparisons between fixed and mobile bearing designs regarding the risk on PPOL tend to show no differences [34,35]. Furthermore, in our series of uncemented LCS patients this is an extremely rare complication (unpublished data).

In our case, PPOL was discovered with conventional radiographs (Fig. 1) and a computed tomography (CT) scan was made with metal suppression (Fig. 2) to further objectify the bone defects for surgical planning. Multiple studies have been done on the accuracy of different imaging modalities to identify, classify and monitor osteolytic bone defects during TKA follow-up [[36], [37], [38], [39], [40], [41]]. It is well established that conventional radiographs lack sensitivity compared to CT or magnetic resonance imaging (MRI) in identifying osteolytic lesions [[38], [39], [40]]. Reish et al. retrospectively reviewed radiographs of CT-proven osteolytic lesions and found that only 17% of the lesions was detected with conventional anteroposterior and lateral radiographs [38]. However, implementing routine CT or MRI scans into regular TKA follow-up would be cost-intensive. Tomosynthesis is a novel imaging modality that uses tomographic technology with specific metal-suppression protocols [41]. Tomosynthesis provides superior sensitivity (85.4% vs 61.5%) and specificity (87.2% vs 64.1%) compared to CT-scans in the detection of osteolytic lesions, and is safe (radiation dose 6% of that of CT-scan) and cost-effective (costs 28% of that of CT-scan) [41]. Clinical follow-up studies should further explore the efficiency and practicality of this promising imaging modality in the detection of PPOL in the routine follow-up after TKA.

Surgical intervention due to PPOL should focus on exchange of the particle generator (mostly the polyethylene bearing), implant anchorage and alignment, and (metaphyseal) bone defects [42]. There have been several attempts to classify metaphyseal bone defects regarding their location, size and containment [43]. The most used classification system is the AORI, which distinguishes 3 different types of bone loss based on size, location and soft tissue [13]. AORI type 1 defects are typically small and contained, recommended treatment is cement filling or allograft bone impaction [43]. AORI 2A and 2B defects are small to moderate and uncontained, and are typically treated with metal augments (dependent on the size of the defect) or allograft bone impaction [43]. Large, uncontained bone defects (AORI type 3) are typically treated with revision components and metal (porous coated) sleeves and cones or morselized impaction bone grafting to fill up large defects and provide mechanical stability [43]. The surgeon should also consider functional demand, patient age and bone quality when choosing between metal sleeves or cones and bone stock reconstruction (i.e. allograft impaction), the latter being reserved to younger patients with higher functional demands and good bone quality [44]. Our case represents a bilateral type 2B metaphyseal bone loss in both femoral condyles, with good anchorage of the prosthesis. According to the recommendations [43], our patient would be best suited with a metal augment or allograft bone impaction due to the size and location of the defect. Since the knee implants in our case demonstrated reliable anchorage to the bone and therefore no revision of femoral components were needed [42], allograft bone impaction deserved the preference. Our case thus shows the possibility of adequate implant anchorage even with the presence of massive osteolytic lesions, demonstrated by the continued survival of the primary tibial and femoral components after 12 years.

## 4 Conclusion

We present an unusual case of bilateral PPOL in the femur and patella after uncemented mobile bearing TKA without patellar resurfacing. During revision surgery, both femoral and tibial components were well fixed to the bone despite the large osteolytic lesions that were found. Synovectomy, polyethylene exchange and morselized allograft bone impaction were the surgical interventions that were undertaken, there were no signs of advanced wear on the metal components or the polyethylene insert. Eight years post revision surgery, the patient remains asymptomatic and there are no new osteolytic lesions detected on radiographic follow-up. There are several (patient and implant) factors that are associated with an increased risk of PPOL (e.g. screw fixation, polyethylene sterilization method, patient age and male gender). Conventional radiographs lack the sensitivity to identify all osteolytic lesions, but implementing CT or MRI in regular TKA follow-up would be too cost-intensive. Surgical intervention in the context of massive PPOL should focus on polyethylene exchange (or exchange of another particle generator), implant anchorage and alignment and bone defects. Dependent on the size and location, these bone defects can be filled up with morselized allograft impaction, metal augments or revision components with porous coated sleeves or cones.

## Conflicts of interest

N/A.

## Funding

N/A.

## Ethical approval

Not needed for this case report.

## Consent

Written informed consent was obtained from the patient for publication of this case report and accompanying images. A copy of the written consent is available for review by the Editor-in-Chief of this journal on request. Consent has been obtained in the native language of the patient (Dutch) since the patients does not understand English. A formal translation of a native English speaker is available upon request.

## Author’s contribution

Rachid Rassir: Conceptualization; Formal analysis; Writing - original draft; Writing - review & editing.

Jorm M. Nellensteijn: Conceptualization; Data curation; Investigation; Writing - original draft; Writing - review & editing.

Rachid Saouti: Conceptualization; Data curation; Resources; Supervision; Writing - original draft; Writing - review & editing.

Peter A. Nolte: Conceptualization; Data curation; Investigation; Supervision; Writing - original draft; Writing - review & editing.

## Registration of research studies

Not applicable.

## Guarantor

Peter A. Nolte.

## Provenance and peer review

Not commissioned, externally peer-reviewed.
